# BMS-265246, a Cyclin-Dependent Kinase Inhibitor, Inhibits the Infection of Herpes Simplex Virus Type 1

**DOI:** 10.3390/v15081642

**Published:** 2023-07-28

**Authors:** Lefang Jiang, Yang Yu, Zhuogang Li, Yarou Gao, Haonan Zhang, Mingxin Zhang, Weihua Cao, Qun Peng, Xulin Chen

**Affiliations:** Guangdong Provincial Key Laboratory of Virology, Institute of Medical Microbiology, Jinan University, Guangzhou 510632, China; lfjiang28@163.com (L.J.); yuyang_0821@163.com (Y.Y.);

**Keywords:** BMS-265246, herpes simplex virus type 1, cyclin-dependent kinase, antiviral, transcription

## Abstract

Herpes simplex virus type 1 (HSV-1) infections are prevalent illnesses that can cause mucocutaneous ulcerative disease, keratitis, and genital herpes. In patients with compromised immune systems, the infection can lead to serious problems, such as encephalitis. Additionally, neonatal infections can cause brain problems and even death. Current first-line antiviral drugs are nucleoside analog inhibitors that target viral polymerase, and resistant strains have emerged. As a result, new drugs with distinct action modes are needed. Recent research indicates that cyclin-dependent kinases (CDKs) are prospective antiviral targets. Thus, CDK inhibitors may be effective antiviral agents against HSV-1 infection. In this study, we examined a panel of CDK inhibitors that target CDKs in the present study. BMS-265246 (BMS), a CDK 1/2 inhibitor, was found to effectively limit HSV-1 multiplication in Vero, HepG2, and Hela cells. A mechanism of action study suggested that BMS inhibits the early stages of viral replication when added early in the viral infection. The suppression of multiple steps in viral replication by BMS was revealed when HSV-1 infected cells were treated at different time periods in the viral life cycle. Our results suggest that BMS is a potent anti-HSV-1 agent and unique in that it may interfere with multiple steps in HSV-1 replication.

## 1. Introduction

Human herpes simplex virus type 1 (HSV-1) is a double-stranded DNA virus belonging to the *Herpesviridae* family, subfamily *Alphaherpesvirinae*, and the genus *Simplexvirus*. It can latently persist in the neurons of infected individuals. Sporadically, HSV-1 can enter a lytic replication cycle through reactivation. HSV-1 is commonly transmitted by oral-to-oral contact and causes feverish blisters or cold sores. However, HSV-1 can also cause genital herpes through oral–genital contact. An estimated 3.7 billion people aged 0 to 49 had HSV-1 infections among the world’s population [1]. HSV-1 infections may be more recurrent and lead to severe complications in immunocompromised patients [2,3,4]. Neonatal infections can cause neurological disorders and death [5,6]. Although uncommon, HSV-1 may cause additional severe complications, such as encephalitis or keratitis [1].

Current antiviral medications, such as acyclovir, famciclovir, and valacyclovir, can reduce the severity and frequency of symptoms but cannot cure the infection [1]. These nucleoside analog inhibitors all target viral polymerase. Several newer agents are undergoing clinical development [7,8,9], but none can entirely suppress herpesvirus infections [8]. For the existing drugs, resistant strains develop and are especially common in immunocompromised individuals [10,11,12,13] and are also significant in patients with ocular infections and in children [11,14,15]. Thus, new drugs with different mechanisms of action are needed.

Despite HSV-1 being a large DNA virus encoding more than 80 genes, it depends on host machinery for virus replication in cell culture and in vivo [16,17,18,19]. CDKs, in addition to being responsible for the progression of the cell cycle from one phase to the next, ensuring that DNA replication and cell division occur in a timely and controlled manner, play an essential role in HSV-1 replication. The dependence of CDKs for HSV-1 replication was largely revealed through anti-HSV-1 research using CDK inhibitors. Schang et al. found that several CDK inhibitors inhibit HSV-1 replication by interfering with the transcription of viral IE, E, and L genes [20,21,22,23,24]. It was found that cdk1, 2, or 7 is required for HSV replication in nonneuronal cells, while cdk2 is required for HSV-1 reactivation in neurons [25]. In addition, it was reported that CDK inhibitors inhibit the replication of many viruses, including HCMV [26], HSV-1 [21,24,27], VZV [28], HIV-1 [29,30,31], influenza A virus (IAV) [32], Zika virus [33], and HBV [34]. Therefore, CDKs are potential antiviral targets for anti-HSV-1, and CDK inhibitors may be potential antiviral agents for HSV-1 infections.

This study aimed to identify novel anti-HSV-1 agents by screening a range of CDK inhibitors that target most CDKs. Then, through a mechanism of action study to find how the newly identified CDK inhibits affect the replication of HSV-1, we found that BMS, a CDK 1/2 inhibitor, demonstrated the ability to effectively inhibit HSV-1 replication in Vero, HepG2, and Hela cells. Further investigation into its mechanism of action revealed that BMS can target multiple stages of HSV-1 replication.

## 2. Materials and Methods

### 2.1. Cell Culture and Virus Infection

African green monkey kidney cells (Vero, ATCC CCL-81), the human hepatocellular carcinoma cell line (HepG2, ATCC HB-8065), the human cervical cancer cell line (HeLa, ATCC CCL-2) were maintained in Dulbecco’s modified Eagle’s medium (DMEM, Corning, NY, USA) supplemented with 10% FBS (Meilunbio, Dalian, China), with 1% penicillin/streptomycin (P/S) (Gibco, Rockville, MD, USA) at 37 °C in an atmosphere containing 5% CO_2_. HSV-1 strain H129 and HSV-2 strain G were obtained from the Wuhan Institute of Virology, Chinese Academy of Sciences. These viruses were propagated in Vero cells and stored at −80 °C until use.

### 2.2. Compounds

BMS-265246, Dinaciclib, Seliciclib, Ro-3306, Abemaciclib, Flavopiridol, LDC000067, NU6300, Purvalanol B, THZ2, Butyrolactone I, CVT-313, BGG463, PNU112455A, and MSC2530818 were purchased from Target Mol (Boston, MA, USA); acyclovir (ACV) was purchased from Solarbio (Beijing, China); Dextran Sulfate Sodium (DSS) was purchased from MP Biomedicals (Santa Ana, CA, USA). Stock solutions were prepared in DMSO (Sigma-Aldrich, St Louis, MO, USA) and stored at −40 °C. For all compounds, the working concentrations were prepared by diluting stock solutions in a culture medium immediately before use.

### 2.3. Antibodies

Anti-HSV-1 + HSV-2 gB mouse monoclonal (ab6506), anti-HSV-1 ICP4 mouse monoclonal (ab6514), anti-HSV-1 ICP8 mouse monoclonal (ab20194), anti-HSV1 + HSV2 gD mouse monoclonal (ab6507), and anti-HSV-1 + HSV-2 ICP5 mouse monoclonal (ab6508) were purchased from Abcam (Cambridge, UK). Horseradish peroxidase (HRP)-conjugated anti-mouse IgG (SA00001-1, Proteintech, Wuhan, China) were used for immunoblotting, and goat anti-mouse 488 Alexa Fluor secondary antibody (4408S, CST, Danvers, MA, USA) was used for immuno-fluorescence. DAPI solution (C0060, Solarbio, Beijing, China) was used to stain the nuclei of the cells.

### 2.4. Cell Viability Assay

For the cell viability assay, cells at a density of 1.5 × 10^4^ cells/well were seeded in 96-well plates. Following treatments with serially diluted compound for 72 h, cell viabilities were measured by a CellTiter-Glo luminescent cell viability assay (Promega, Madison, WI, USA), based on quantification of the ATP content, according to the manufacturer’s protocol. Luminescence signals were detected using a Varioskan LUX multimode microplate reader, and cell viability was normalized to the vehicle-treated cell. Experiments were performed in a triplet for each condition.

### 2.5. Plaque Assay

Monolayers of Vero cells (4 × 10^5^ cells/well) in 12-well plates were infected with serially diluted virus samples and incubated for 2 h at 37 °C. The inoculum was removed; cells were washed with PBS and overlaid with maintenance DMEM containing a final concentration of 1.5% cellulose colloidal, 2% FBS, 100 U/mL of penicillin, and 100 μg/mL of streptomycin. After incubation for two days at 37 °C with 5% CO_2_, cells were fixed with 4% paraformaldehyde, followed by staining with 0.5% crystal violet in 20% ethanol for plaque counting.

### 2.6. Antiviral Activity Assays

The antiviral activity in the primary screening was evaluated using a cytopathic effect (CPE) reduction assay [35]. Briefly, Vero cells in 96-well plates were infected with HSV-1 (MOI = 0.01) and then treated with compounds at indicated concentrations in triplicate. After 48 h of incubation, the cells were fixed at room temperature for 30 min with 4% formaldehyde. After the removal of the formaldehyde, the cells were stained with 0.1% (*w*/*v*) crystal violet for 30 min at room temperature. The plates were gently rinsed and dried, and the intensity of the crystal violet stain was measured at 570 nm for each well. The concentration required for a test compound to reduce the CPE caused by HSV-1 infection by 50% (IC_50_) was determined.

Antiviral activity in secondary screening was assessed based on viral titers determined using a plaque reduction assay. Vero cells in 96-well plates were infected with HSV-1 (0.01 MOI) and added compounds at indicated concentrations in triplicate. After 48 h of infection, viral titer in the supernatant was detected by plaque assay. The concentration of the test compound required to reduce the plaques formed by HSV-1 infection by 50% (IC_50_) was determined.

### 2.7. Time-of-Addition Assay

Time-of-addition assay was performed as previously described [35]. Briefly, Vero cells were infected with HSV-1 (MOI = 0.01) under different treatment settings. (a) Pretreatment of cells: Vero cells were pretreated with 16 μM of BMS before infection; (b) attachment: Vero cells were infected in media containing 16 μM of BMS at 4 °C for 1 h; (c) post-adsorption: after removal at 1, 4, and 10 h post-infection of HSV-1, 16 μM of BMS was added to the cells. Cells were cultured at 37 °C for 48 h, and virus yields in the supernatants were determined by plaque assay.

### 2.8. Indirect Immunofluorescence Assay

The treated cells were washed with PBS on a confocal dish. Cells were fixed with 4% paraformaldehyde (PFA) for 15 min and then incubated in 0.2% Triton X-100 for membrane permeabilization for 10 min at room temperature. Cells were blocked with 5% BSA in PBS for 1 h and with primary antibodies overnight at 4 °C. Next, the cells were washed with PBS three times and incubated with Alexa Fluor 488 labeled secondary antibody for 1 h at 37 °C. Immunofluorescence images were taken with a Leica SP8 confocal microscope.

### 2.9. Virus Attachment Assay

The virus attachment assay was performed using IFA. 6 × 10^4^ cells/dish Vero cells were cultured on glass bottom dishes (NEST, Wuxi, China). After one hour of pre-cooling, the cells were infected with HSV-1 at an MOI of 10 in the presence of compounds at indicated concentrations and incubated at 4 °C for 2 h. Then, the cells were fixed, permeabilized, blocked, and stained with anti-gD antibody, subsequently stained with Alexa Fluor 488 labeled secondary antibody, followed by incubation with DAPI using IFA. Images were captured using a Lecia laser scanning microscope.

### 2.10. Virus Entry Assay

The virus entry assay was performed using IFA. Vero cells at a density of 6 × 10^4^ cells/dish were cultured in laser confocal dishes. Vero cells were pre-cooled for 1 h at 4 °C, then infected with HSV-1 (MOI = 10) for 1 h at 4 °C. The unadsorbed viruses were removed by washing with PBS. Then, cells were treated with the compound for 30 min at 37 °C to maximize virus penetration and washed with low-acidic PBS (pH3) for 60 s to remove the viruses on cell membranes. The cells were then fixed, permeated, blocked, and stained with an anti-ICP5 antibody, which was subsequently stained with Alexa Fluor 488 labeled secondary antibody, followed by incubation with DAPI using IFA. The images were captured using a Lecia laser scanning microscope.

### 2.11. Western Blot

Cells were collected and lysed in RIPA buffer (Beyotime, Shanghai, China) and incubated on ice for 30 min. Lysates were centrifuged at 4 °C for 10 min at 12,000× g, and supernatants were collected. Protein concentrations were determined using a Pierce BCA protein assay kit (Biosharp, Hefei, China). The proteins were resolved by SDS-PAGE and transferred to a PVDF membrane. TBS-T supplemented with 5% non-fat dry milk (*w*/*v*) was used for blocking for 1 h, and then the membranes were incubated at 4 °C overnight with primary antibodies. Blots were rinsed three times for 5 min each and then incubated with the secondary antibodies (1:2000–3000 dilutions) in 3% non-fat milk in TBS-T at room temperature for 1 h. The ECL reagents were used for detection. The signal was captured with a ChemiDoc^TM^ imaging system (Bio-Rad, Singapore, Singapore).

### 2.12. Quantitative Real-Time PCR of Viral mRNA and DNA

Vero cells (1.2 × 10^6^ cells/well) were infected with HSV-1 at an MOI of 0.01 in 6-well plates, then BMS with final concentrations of 50 μM, 5 μM, and 0.5 μM were added and cultured at 37 °C. After 24 h, each supernatant viral DNA was extracted and purified using a Virus DNA/RNA Magnetic Beads Kit (M502-01, Genfine, Beijing, China). The intracellular viral DNAs were extracted using the Hirt method, and the total intracellular RNAs were extracted using the Trizol method. Finally, a quantitative analysis was performed using real-time PCR.

Extrachromosomal DNAs in cells were isolated using a modified Hirt method. Briefly, cells were lysed in Hirt lysis buffer (0.6% SDS, 10 mM Tris pH 7.5, 10 mM EDTA pH 8.0) and treated with proteinase K (50 µg/mL) (Genfine, Beijing, China). Then, sodium chloride was added to a final concentration of 1 M, and the mixture was incubated overnight at 4 °C. The high molecular weight (chromosome) DNAs and proteins were precipitated and discarded. The viral DNAs were then purified by phenol extraction and precipitated with 0.3 M sodium acetate and 70% ethanol. The precipitated DNA was washed and resuspended in DNAase-free water.

The total RNAs of Vero cells were isolated using Trizol reagent (Invitrogen), and cDNA was then generated by reverse transcription from 1 μg total RNA using a Re-verse Transcriptase Kit (M-MLV) (Zoman Biotechnology, Beijing, China). Quantitative real-time PCR was carried out using Magic SYBR Mixture (CWBIO, Beijing, China). The primers used for real-time PCRs are listed in Table 1.

### 2.13. Statistical Analyses

**Figure 1:**
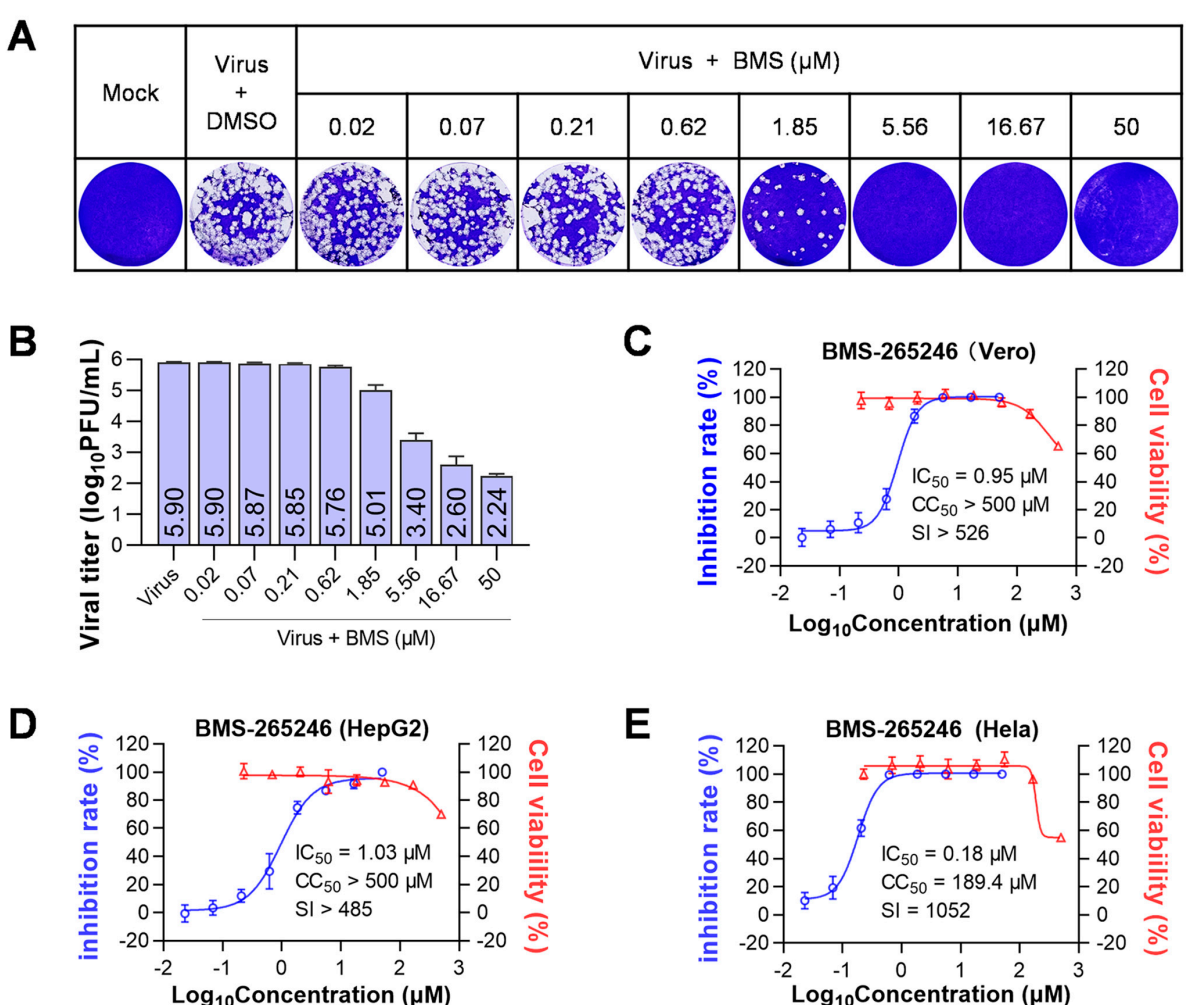
BMS inhibits the replication of HSV-1 in vitro. Cells were infected with HSV-1 (MOI = 0.01) and treated with serially diluted BMS (starting at 50 μM and diluted in a triple gradient) at 37 °C. After 48 h of incubation, supernatants were collected, and viral titers were determined by plaque assay. The cell viability of Vero, HepG2, and HeLa cells was determined by Cell Titer-Glo assay after 72 h exposure to BMS at the indicated concentration. (**A**) The production of progeny viruses in HSV-1 infected cells treated at the indicated drug concentrations was detected by plaque assay. (**B**) The chart depicts the virus titers in the supernatants of HSV-1-infected cells treated with varying amounts of BMS by counting the visible plaques, as shown in (**A**). Values are mean ± standard deviation (*n* = 3). The kinetics of anti-HSV-1 activity and cytotoxicity of BMS were analyzed in Vero (**C**), HepG2 (**D**), and Hela (**E**) cells.

Data in the primary and secondary screen (Table 2 and Figure 1) were obtained according to the dose-response curves fitted using nonlinear regression, log [drug] vs. response, and variable slope (four parameters) in GraphPad Prism v8.0.0 (GraphPad Software, San Diego, CA, USA). The selective index (SI) was calculated as the ratio of the CC_50_ (the concentration required to reduce cell viability by 50%) to the IC_50_ (the concentration needed to inhibit cytopathicity by 50%). Differences between experimental groups were statistically analyzed using Student’s *t*-test, with *p*-values < 0.05 being statistically significant. Symbols for *p*-values used in the figures: * *p* < 0.05, ** *p* < 0.01, *** *p* < 0.001, and **** *p* < 0.0001.

## 3. Results

### 3.1. BMS Was Identified to Inhibit the Replication of HSV-1 and HSV-2 In Vitro

To initiate a primary screening of CDK inhibitors for anti-HSV-1 agents, we selected 15 compounds that target one or multiple CDKs: CDK1, CDK2, CDK4, CDK5, CDK6, CDK7, CDK8, and CDK9. Using a CPE reduction assay on Vero cells infected with HSV-1, we screened these CDK inhibitors at concentrations ranging from 0.006 to 100 μM for anti-HSV-1 activity. Among the 15 CDK inhibitors, BMS, a CDK1/2 inhibitor, and MSC2530818 (MSC), a CDK8 inhibitor, were identified to reduce CPE caused by HSV-1 infection in a dose-dependent manner. The IC_50_s for BMS and MSC were determined to be 0.08 µM and 25 µM, respectively. The CC_50_s for BMS and MSC were determined to be >500 µM and 138.5 µM, respectively. Therefore, the selective indices for BMS and MSC were >6250 and 5.54, respectively. In contrast, other CDK inhibitors exhibit more significant cytotoxicity and no antiviral activity (Table 2). These results suggest that BMS has potent anti-HSV-1 activity on Vero cells.

To assess the antiviral effect of BMS, we measured in vitro the production of infectious HSV-1 virions on HSV-1-infected Vero cells treated with serially diluted compounds by plaque assay. Our results showed that BMS has a dose-dependent anti-HSV-1 effect and an IC_50_ of 0.95 µM. Additionally, BMS has low cytotoxicity, with a CC_50_ of over 500 μM (Figure 1A–C).

In addition, we tested the antiviral activity of BMS on HepG2 and Hela cells. The results showed that BMS had IC_50_s of 1.03 µM and 0.18 µM on HepG2 and Hela cells, respectively. Additionally, the CC_50_s for BMS were determined to be >500 µM and 189.4 µM on HepG2 and Hela cells, respectively. The selective index was >485 for HepG2 cells and 1052 for Hela cells, indicating that BMS is more effective on Hela cells than Vero and HepG2 cells (Figure 1D,E).

Next, BMS was tested for its anti-HSV-2 activity in Vero cells based on the production of infectious virions as determined by plaque assay. The IC_50_, CC_50_, and selective index were determined to be 1.06 μM, >500 µM, and >472, respectively. BMS has a similar antiviral effect against HSV-2 to that against HSV-1 in Vero cells.

Thus, out of the 15 CDK inhibitors tested, BMS is identified as the most effective in inhibiting the replication of both HSV-1 and HSV-2 in vitro.

### 3.2. BMS Inhibits the Early Stage of HSV-1 Replication

BMS is a potent CDK1/CDK2 selective inhibitor [36]. It was reported that one or more CDKs are required for the accumulation of HSV transcripts, viral DNA replication, and the production of infectious viruses [20]. To understand how BMS inhibits HSV-1 replication, we did a time-of-addition assay to elucidate which stage of the HSV-1 life cycle is affected.

**Figure 2:**
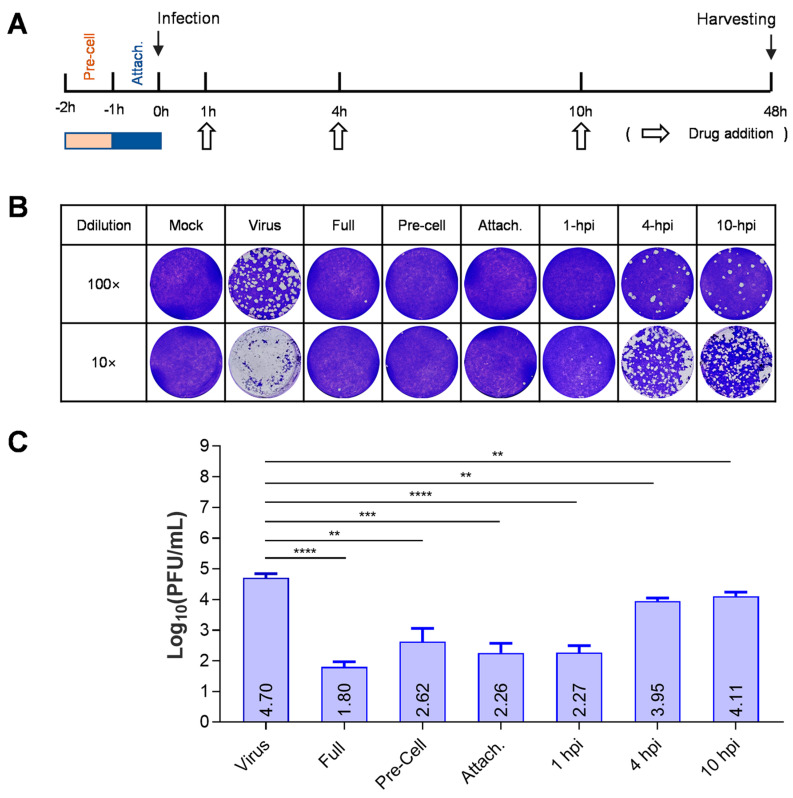
BMS suppresses the early stage of HSV-1 replication. (**A**) Schematic diagram of a “time-of-addition assay” to determine the stage of HSV-1 replication inhibited by BMS. (**B**,**C**) Time-of-addition assay. Vero cells were infected with HSV-1 and treated with 16 μM BMS started at different time points post-virus infection. At 48 hpi, the antiviral effect of BMS was detected by plaque assay. The representative images (**B**) and quantitative results (**C**) are shown. Data are presented as mean ± SD of three independent experiments. Symbols for *p*-values used in the figures: ** *p* < 0.01, *** *p* < 0.001, and **** *p* < 0.0001.

The effect of each treatment (time-of-addition) on the production of infectious virions of HSV-1 was evaluated using a plaque assay. We found that the production of virions at 48 h post-infection (hpi) was significantly inhibited with the pretreatment of cells, adding drugs during attachment or at 1 hpi (Figure 2). Compared with a viral titer of 10^4.7^ on untreated cells, viral titers in the supernatant of treated cells were reduced to 10^1.8^, 10^2.26^, and 10^2.27^, respectively, with treatment beginning at the beginning of viral infection and ended until harvesting, treatment during attachment, and treatment beginning at 1 hpi and completed until harvesting. When the treatment started at 4 or 10 hpi and finished until harvesting, the viral titer reduction was less than one logarithm. Interestingly, pretreatment of the cells also significantly reduced the production of progeny virions. Together, these results indicate that BMS suppresses the early stage of HSV-1 replication.

### 3.3. BMS Does Not Inhibit HSV-1 Attachment to Host Cells

**Figure 3:**
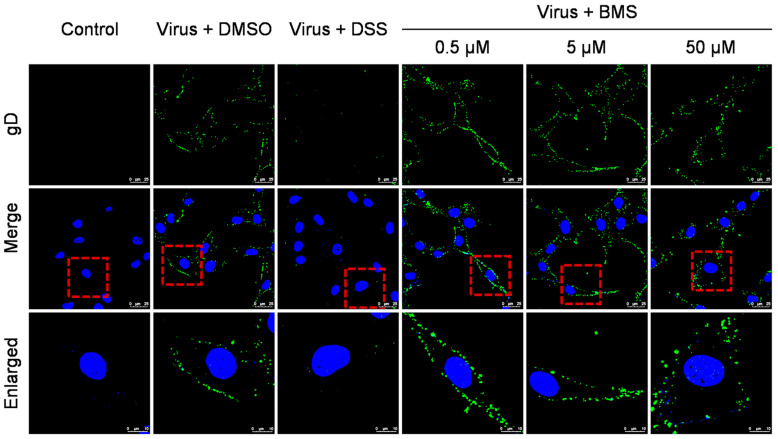
BMS does not inhibit the attachment of HSV-1 to host cells. The effect of BMS on viral attachment was assessed using IFA. The cell monolayers were pre-chilled at 4 °C for 1 h and then were infected with HSV-1 at an MOI of 10 in the presence of BMS at indicated concentrations and incubated at 4 °C for 2 h. Then, the cells were fixed, permeabilized, blocked, and stained with DAPI (blue) and gD (green) using IFA. Representative images from the three independent experiments obtained with a Leica SP8 microscope are shown. The magnification is 63× under oil immersion. For better visibility, the red dotted line selected area is enlarged and placed in the image below.

The binding of the virus to the host cell membrane is the first step in viral infection. We performed an attachment assay to check if BMS affects the attachment of HSV-1 to host cells. In order to prevent the virus membrane from fusion with the host cell membrane, the assay was performed at 4 °C. As shown in Figure 3, the treatments of BMS at 0.5, 5, and 50 µM did not affect the binding of HSV-1 to Vero cells. Whereas the reference compound DSS, which was reported to inhibit the attachment of many viruses such as HIV [37], SARS-CoV-2 [38], and influenza virus [39], completely inhibited the binding of HSV-1 to Vero cells at a concentration of 100 µg/mL. Thus, BMS does not suppress HSV-1 attachment to host cells.

### 3.4. BMS Does Not Inhibit the Entry of HSV-1 into Host Cells

**Figure 4:**
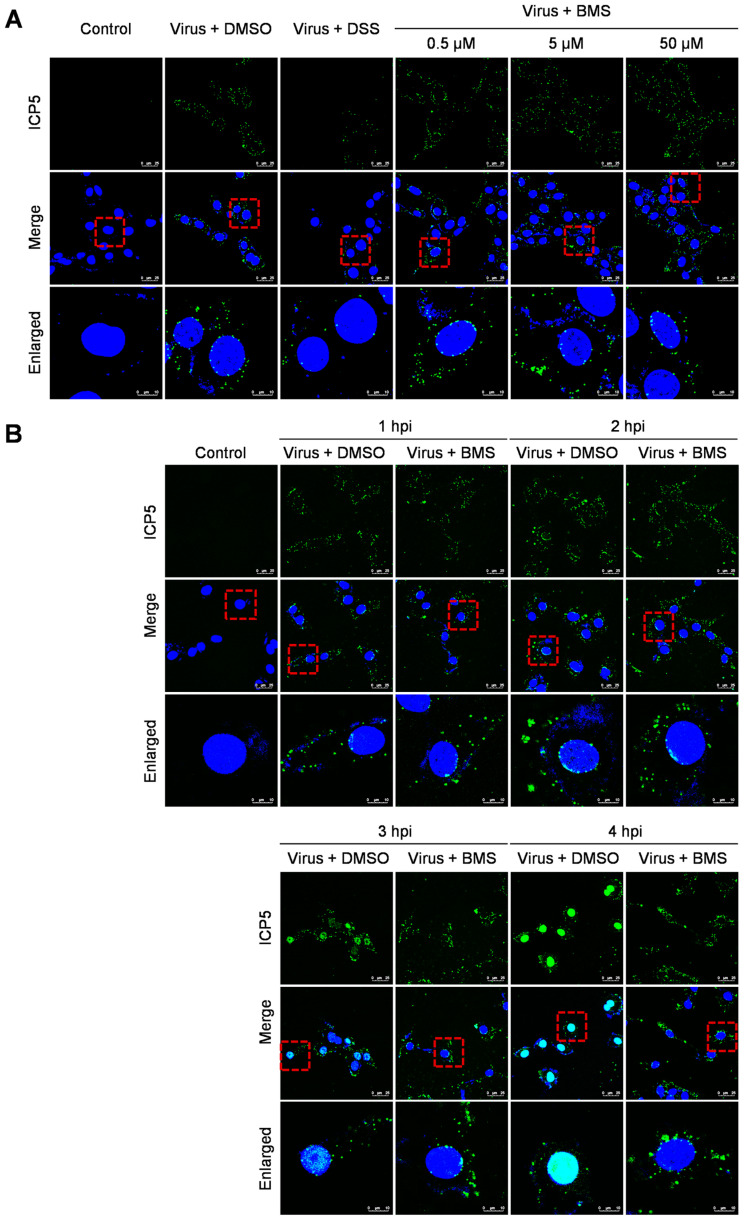
BMS does not interfere with the entry of HSV-1. (**A**) The Vero cell monolayers were pre-chilled at 4 °C for 1 h, then incubated with the virus of 10 MOI at 4 °C for another 2 h to allow virus attachment. Then, BMS or DSS at the indicated concentration was added. The culture was incubated at 37 °C for 30 min to maximize virus penetration. The infected cell monolayers were treated with acidic PBS (pH 3) for 60 s to inactivate the non-penetrated viruses and washed twice with PBS. (**B**) Vero cells were infected with 10 MOI HSV-1 in the presence or absence of BMS or positive drug DSS for 1, 2, 3, and 4 h at 37 °C. The cells were fixed, permeated, and prepared for immunofluorescence assays. Antibody against ICP5 (green) was used, and nuclei were stained with DAPI (blue). Representative images from three independent experiments obtained with a Leica SP8 microscope are shown. The magnification is 63× under oil immersion. The red dotted line selected area is enlarged and placed in the image below for better visibility. The cyan color resulted from the merge of green (representing ICP5) and blue (representing the DAPI-stained nucleus).

HSV-1 enters the host cell via the fusion of the viral envelope with the cytoplasmic membrane and further transport of the viral capsid to the nucleus. Next, we performed an entry assay to determine whether the entry of HSV-1 was blocked by the BMS. After binding at 4 °C, Vero cells were cultured at 37 °C for 30 min to allow entry of the viruses. The localization of viral capsid was determined by indirect immunofluorescence assay (IFA) using a primary antibody against the major capsid protein ICP5. As shown in Figure 4A, the treatments of BMS at 0.5, 5, and 50 µM did not affect the entry of HSV-1 to Vero cells. The viral capsids are in the cytoplasm, some distributed around the nuclei. However, we have not been able to determine whether the viral genome entered the nuclei by visualization of the viral genome through fluorescence in situ hybridization (FISH). Instead, we examined the localization of ICP5 over time to determine whether the viral genome entered the nucleus and initiated the transcription and translation of the viral gene ICP5. As shown in Figure 4B, the viral capsid entered the cytoplasm and perinucleus outside the nucleus at 1 hpi, and the new ICP5 was synthesized from 3 hpi and reached a high level at 4 hpi. When the cells were treated with BMS, the virus could still attach to the cell and enter its cytoplasm. However, the expression of ICP5 was prevented. This suggests that BMS does not interfere with the attachment and entry of HSV-1 into the host cell, but it may suppress the release of the viral genome into the nucleus or, more likely, the transcriptional function of the host cell.

### 3.5. The Expression of HSV-1 IE, E, and L Genes Are Affected by BMS Treatment

In the life cycle of HSV-1, the immediate early genes (IE), early genes (E), and late genes (L) are expressed (transcribed and translated) in a sequentially ordered cascade. The IE gene is expressed first, followed by the E gene expression and DNA replication. The L gene is lastly expressed. We evaluated the effect of BMS treatment on viral gene transcription and translation.

**Figure 5:**
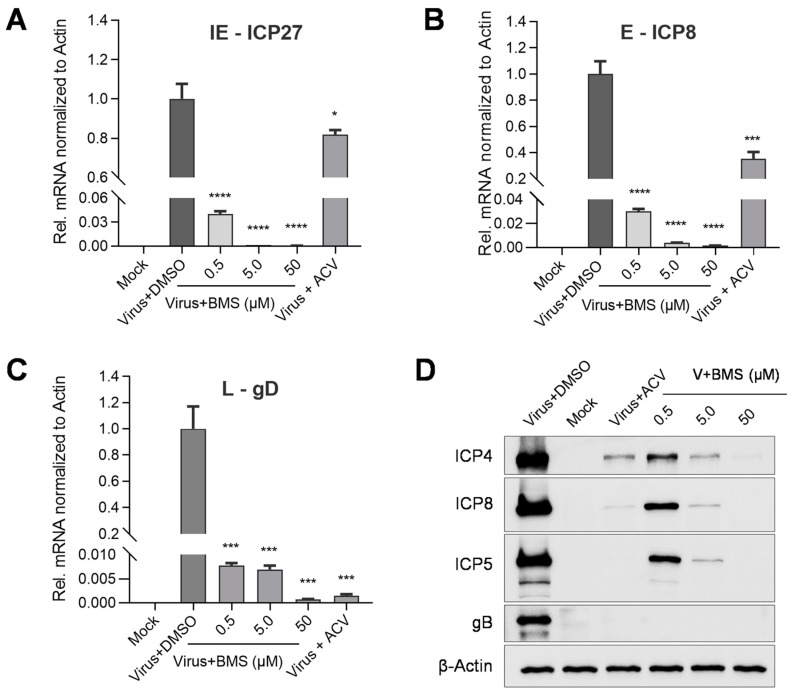
BMS inhibits the expression of HSV-1 IE, E, and L genes. Vero cells were infected with HSV-1 (MOI = 0.01) in the presence of BMS or ACV (10 µM). Then, the total RNAs of HSV-1 ICP27, ICP8, and gD were extracted at 4, 8, and 20 hpi, respectively. The levels of ICP27 (**A**), ICP8 (**B**), and gD (**C**) were analyzed by qRT-PCR. (**D**) For the detection of viral proteins, total cellular proteins were extracted at 24 hpi. The ICP4, ICP8, ICP5, and gB protein levels were evaluated by Western blotting. Data are mean ± SD (*n* = 3). Symbols for *p*-values used in the figures: * *p* < 0.05, *** *p* < 0.001, and **** *p* < 0.0001.

As shown in Figure 5, the transcripts of the IE gene ICP27, the E gene ICP8, and the L gene gD were BMS concentration-dependently suppressed. The protein levels of IE protein ICP4, E protein ICP8, and two L proteins, ICP5, and gB (Figure 5D), were also reduced by BMS concentration-dependently. The production of infectious virions was also reduced concentration-dependently in the antiviral experiments (Figure 1).

Altogether, these results suggest that BMS treatment reduces HSV-1 gene transcription, translation, and virion production, again suggesting that BMS may hinder the release of the viral genome into the nucleus or the transcriptional function of the host cell.

### 3.6. The Intracellular and Virion-Associated HSV-1 Viral DNA Levels Are Decreased upon BMS Treatment

**Figure 6:**
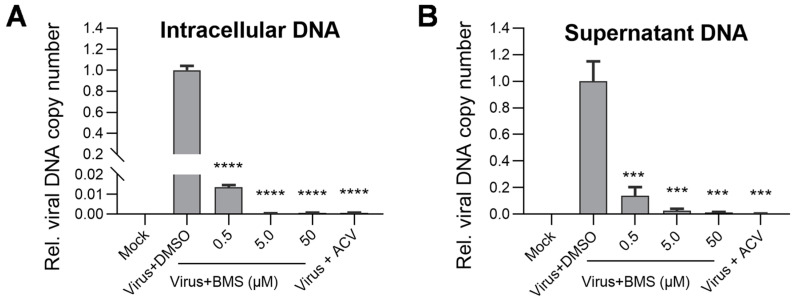
The production of intracellular viral DNA levels and virion-associated DNA levels were decreased upon BMS treatment. Vero cells were infected with HSV-1 (MOI = 0.01) in the presence of BMS, or ACV (10 µM), and cultured for 24 h. Then, the intracellular HSV-1 DNA was extracted by the Hirt method, and qPCR was performed to measure viral DNA amounts (**A**). In parallel, the supernatant virion-associated DNAs of HSV-1 were extracted using a Virus DNA/RNA Kit (Magnetic Beads) (GENFINE Biotech, Beijing, China), and qPCR was performed to measure viral DNA amounts (**B**). The results shown are means ± SD from three independent experiments. Statistical analysis was performed using the Student’s *t*-test; *** *p* < 0.001 and **** *p* < 0.0001.

DNA replication is an integral step in the HSV-1 life cycle that relies on its own machinery, including an origin-binding protein (UL9), single-stranded DNA binding protein (ICP8), DNA polymerase (UL30), processivity factor (UL42), and a helicase/primase complex (UL5/UL8/UL52). These are proteins encoded by the E genes of HSV-1. To assess the effect of BMS on HSV-1 DNA replication, we determined intracellular and virion-associated HSV-1 DNA levels by real-time PCR. Our results show that the viral DNA replication represented by the intracellular HSV-1 DNA levels decreases concentration-dependently (Figure 6A). Similarly, the virion-associated HSV-1 viral DNA levels are also reduced concentration-dependently (Figure 6B). These observations suggest that BMS blocks the replication of HSV-1 DNA and the production of progeny virions, possibly due to the transcription of viral genes being inhibited by BMS.

### 3.7. BMS Suppresses the Transcription of IE, E, and L Genes of HSV-1 during Multiple Stages of Virus Replication

**Figure 7:**
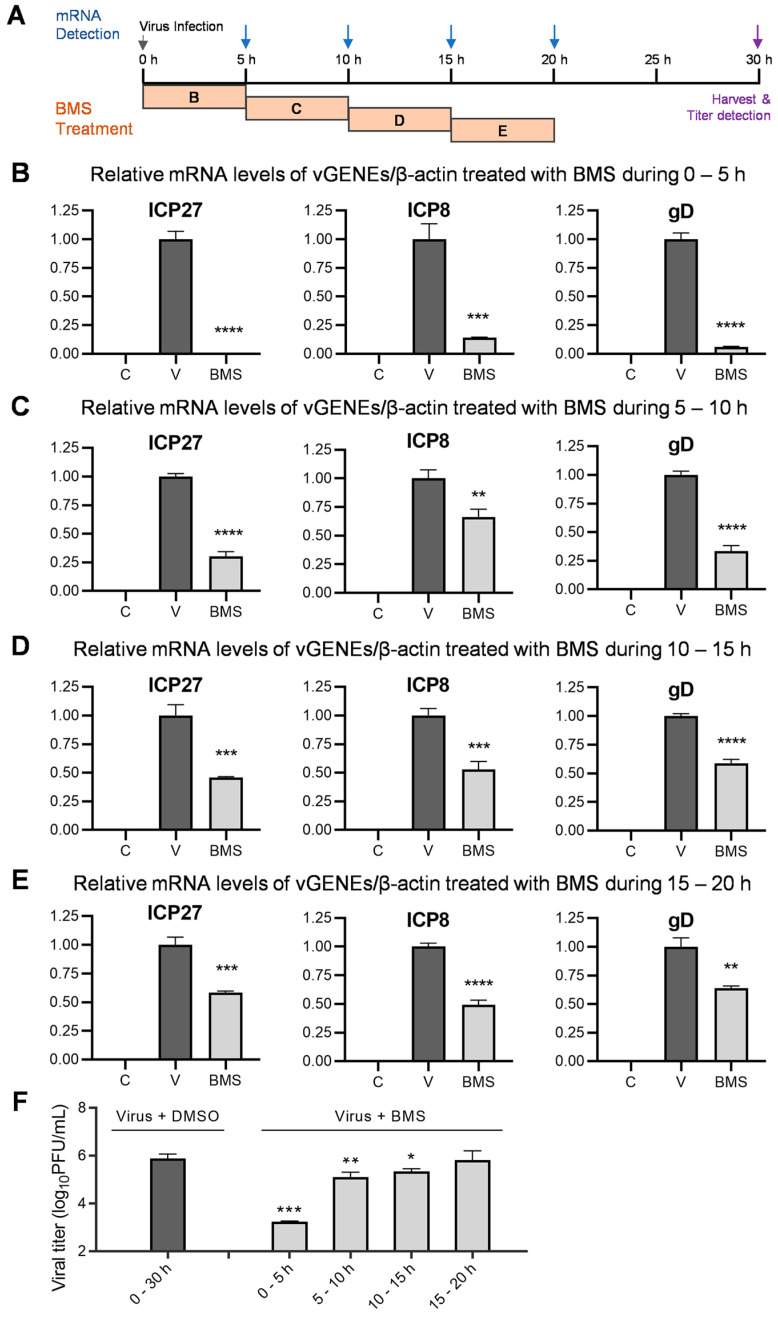
BMS suppresses transcription of the HSV-1 IE, E, and L genes when treated during multiple stages of viral replication. (**A**) Schematic diagram of a BMS treatment during different time periods and when the mRNAs are harvested and detected. (**B**–**E**) Vero cells were mock-infected, or infected with HSV-1 (MOI = 1), and treated with 50 μM of BMS during 0–5 (**B**), 5–10 (**C**), 10–15 (**D**), and 15–20 (**E**) hpi, or left untreated. At the end of each period, at 5 (**B**), 10 (**C**), 15 (**D**), and 20 (**E**) hpi, the relative mRNA levels of ICP27, ICP8, and gD of HSV-1 normalized to β-actin were determined by quantitative real-time PCR analysis. (**F**) Vero cells were infected with HSV-1 (MOI = 0.1) and treated with 50 μM of BMS during 0–5, 5–10, 10–15, and 15–20 hpi. After 30 h of infection, viral titers in the supernatant were detected by plaque assay. The results shown are means ± SD from three independent experiments. Symbols for *p*-values used in the figures: * *p* < 0.05, ** *p* < 0.01, *** *p* < 0.001, and **** *p* < 0.0001. In B, C, D, and E, letter C, mock-infected; letter V, virus-infected; BMS, virus-infected and treated with BMS.

Because the first stage in which BMS acts is between the release of the viral genome into the nucleus and the transcription of the IE genes, the inhibitory effect of BMS, when added early in the course of HSV-1 infection on the later stages, cannot be observed. To answer whether the later stages in HSV-1 replication are affected by BMS treatment, we measured the effect of BMS treatment over four different time periods, 0–5, 5–10, 10–15, and 15–20 hpi, on the transcription of the IE, E, and L genes of the HSV-1 determined at 5, 10, 15, and 20 hpi. In addition, the production of infectious virions on cells treated with BMS during these time periods was determined by plaque assay (Figure 7A). Though as expected, the virus productivity was reduced much more drastically when the HSV-1 infected cells were treated with BMS earlier (during 0–5 hpi, Figure 7F). BMS treatment at 5–10 and 10–15, during which the IE and E genes have already been transcribed, can significantly suppress virus production (Figure 7F). Surprisingly, we found that BMS treatment during all four quarter periods of the 20 h life cycle of HSV-1 dramatically suppresses the transcription of ICP27, ICP8, and gD (Figure 7B–E). These observations indicate that BMS can act on multiple steps in HSV-1 replication by affecting the transcripts of the IE, E, and L genes, presumably through interference with the host transcriptional function.

## 4. Discussion

CDKs play a central role in the cell cycle, and their dysregulation is associated with a number of cancers. HSV-1, such as all other viruses, relies on host-factor-supported functions in gene transcription and translation, DNA replication, and many other activities required for the cell cycle. Similarly, CDKs have been implicated as potential anti-cancer and antiviral targets. Small molecule inhibitors of CDKs that have been developed as potential therapies for cancer may also be effective against viral infections.

In this study, BMS was identified to inhibit HSV-1 replication in 15 CDK inhibitors well below toxic concentrations. MSC, on the other hand, despite its antiviral activity, its IC_50_ is close to CC_50_ and, in its current form, has limited potential for anti-HSV-1 therapy due to its high cytotoxicity. It is important to note that all other CDK inhibitors have considerably higher cytotoxicity than BMS. The CC_50_s are in the lower micromolar range. We could not detect the antiviral activity of other CDK inhibitors against HSV-1 because cytotoxicity interfered with the detection of its antiviral activity. Thus, in addition to identifying a novel anti-HSV agent, our pilot antiviral screening of anti-HSV-1 agents suggests that some CDK inhibitors are considerably less toxic to host cells than others.

To investigate the antiviral mechanism of BMS, we determined the effect of BMS on each stage of the virus life cycle. Through time-of-addition assay, attachment, and entry assays, analysis of the transcription and translation of the viral IE, E, and L genes, the viral DNA replication, and the production of infectious virions, we found that BMS acts in the early stage of the virus life cycle when applied early during viral infection. The first step of HSV-1 replication on which BMS acts is either the release of the viral genome into the nucleus or the transcription of the IE genes. Notably, the suppression of multiple steps in viral gene transcription by BMS was revealed when treated at different time periods in the viral life cycle. Our results suggest that BMS is not only a potent anti-HSV-1 agent with minimal cytotoxicity but also unique in that it may interfere with multiple steps in the HSV-1 replication cycle.

CDK1, or cell division cycle protein 2 homolog, is a highly conserved serine/threonine protein kinase in cell cycle regulation. Approximately 75 targets of CDK1 have been identified that control critical cell cycle events, such as DNA replication and segregation, transcriptional programs, and cell morphogenesis [40]. CDK2, also known as cell division protein kinase 2, is a member of the cyclin-dependent kinase family of Ser/Thr protein kinases. It is highly similar to CDK1 [41]. It is a catalytic subunit of the cyclin-dependent kinase complex, whose activity is restricted to the G1-S phase of the cell cycle, where cells make proteins necessary for mitosis and replicate their DNA [42,43]. BMS is a potent CDK1/CDK2 selective inhibitor with an in vitro IC_50_ of 6 and 9 nM to inhibit cyclin B-dependent CDK1 and cycle E-dependent CDK2 respective by the Bristol-Myers Squibb Pharmaceutical Research Institute in 2003 [36].

BMS inhibits CHI3L1 (chitinase 3-like-1) stimulation of ACE2 (angiotensin-converting enzyme 2) and SPP (viral spike protein priming proteases) and can be potentially used for COVID-19 therapy [44]. Clinical administrations of BMS in cancer therapy suggest it is quite tolerant [45,46]. Because HSV-1 infection or reactivation is acute, antiviral therapy requires only short-term medication. It is expected to be safe in anti-HSV-1 therapy, either topical or systemic administration. If BMS is confirmed to inhibit HSV-1 replication by targeting CDK1/2, the emergence of resistance is unlikely. In these respects, BMS has great potential as an anti-HSV drug. Our study provides additional evidence that CDK1/2 may be useful as an anti-HSV target.

Using either pharmacological inhibition or genetic silencing of CDK1 and CDK2, HCMV-induced SAMHD1 phosphorylation is CDK2-dependent. CDK1 inhibitor CGP74514A treatment dramatically reduced HCMV replication by ~300-fold compared with untreated controls [47]. It was reported that CDK1/2 is required to accumulate HSV transcripts, replicate viral DNA, and produce infectious viruses [20]. Although HSV-1 is a large DNA virus encoding more than 80 genes, it depends on cellular machinery for virus replication and the production of infectious virions in cell culture and in vivo [16,17,18]. Considering that CDK1/2 phosphorylates at least 75 target proteins, and the first BMS targeted function confirmed in this study is the transcription of HSV-1 genes, we assume that BMS inhibits HSV-1 replication by affecting the transcript of IE, E, and L genes, presumably through one or more host factors relating to host transcription function. Nonetheless, additional mechanism-of-action studies focusing on target identification and molecular mechanisms associated with the inhibition of viral gene transcription are needed.

## Figures and Tables

**Table 1:** Primer sequences for the RT-PCR.

Gene	Primer	Sequence
HSV-1 ICP27	Forward	ATCGCACCTTCTCTGTGGTC
	Reverse	GCAAATCTTCTGGGGTTTCA
HSV-1 gD	Forward	TACAACCTGACCATCGCTTG
	Reverse	GCCCCCAGAGACTTGTTGTA
HSV-1 ICP8	Forward	GTCGTTACCGAGGGCTTCAA
	Reverse	GTTACCTTGTCCGAGCCTCC
β-actin	Forward	CTCCATCCTGGCCTCGCTGT
	Reverse	GCTGTCACCTTCACCGTTCC

**Table 2:** Identification of antiviral agents against the infection of HSV-1 among CDK inhibitors.

CDK Inhibitor	Target(s)	CC_50_ (µM)	IC_50_ (µM)	Selective Index
Abemaciclib	CDK4/6	29.11	ND	-
BGG463	CDK2	18.46	ND	-
BMS-265246	CDK1/2	>500	0.08	>6250
Butyrolactone I	CDK1	151.20	ND	-
CVT-313	CDK2	6.38	ND	-
Dinaciclib	CDK1/2/5/9	1.13	ND	-
Flavopiridol	CDK1/2/4	0.16	ND	-
LDC000067	CDK9	14.37	ND	-
MSC2530818	CDK8	138.50	25	5.54
NU6300	CDK2	13.83	ND	-
PNU112455A	CDK2/5	156.40	ND	-
Purvalanol B	CDK2/5	73.52	ND	-
Ro-3306	CDK1/2	14.11	ND	-
Seliciclib	CDK2/5/7	18.47	ND	-
THZ2	CDK7	0.42	ND	-
Acyclovir (Reference)	Herpesvirus POL	>100	0.42	>238.10

Vero cells were infected with HSV-1 at an MOI of 0.01 and treated with CDK inhibitors or acyclovir for 48 h at 37 °C. The anti-HSV effects were evaluated by cytopathic effect (CPE) reduction assay. The data are expressed as means ± SD from three independent experiments. Note: ND: Not detectable. CC_50_: The half maximal cytotoxic concentration. IC_50_: The half maximal inhibitory concentration. SI: Selective Index, SI = CC_50_/IC_50_.

## Data Availability

The datasets generated and/or analyzed in the current study are available from the corresponding author on reasonable request.
